# Clinical and radiological outcome of Mason-Johnston types III and IV radial head fractures treated by an on-table reconstruction

**DOI:** 10.1186/s13018-022-03394-w

**Published:** 2022-11-19

**Authors:** Tobias Kastenberger, Peter Kaiser, Anna Spicher, Kerstin Stock, Stefan Benedikt, Gernot Schmidle, Rohit Arora

**Affiliations:** Department of Orthopaedics and Traumatology, Medical University Innsbruck, Anichstr. 35, 6020 Innsbruck, Austria

**Keywords:** Radial head fracture, On-table, Mason, Reconstruction, Treatment, Surgery, Arthroplasty, Radial head resection

## Abstract

**Background:**

Only few methods treating comminuted radial head fractures have been established providing sufficient joint reconstruction, restoring radial length and enabling early joint mobilization. When an anatomical reconstruction using open reduction and internal fixation is not possible, radial head resection or primary arthroplasty is often conducted. An “Ex situ/on-table” reconstruction is widely disregarded but can be an option. The purpose of this study was to evaluate the functional and radiological outcome of comminuted radial head fractures treated with an “on-table” reconstruction and internal fixation using a low profile plate.

**Methods:**

Fourteen patients who sustained a radial head fracture (9 Mason-Johnston type III and 5 Mason-Johnston type IV) and were treated with an “on-table” reconstruction between 2010 and 2020 were evaluated retrospectively. The patients mean age was 41.3 years (range 21–69). The clinical evaluation included active range of motion, grip strength, pain level and elbow stability. The functional outcome was assessed using the Disability of Arm, Shoulder and the Hand (DASH) score, Mayo Elbow Performance Index (MEPI), Broberg and Morrey score. The radiological examination included a.p. and lateral views of the injured elbow to evaluate nonunions, loss of reduction, joint alignment, avascular radial head necrosis, heterotopic ossifications and posttraumatic osteoarthritis.

**Results:**

The inclusion rate was 74% with a mean follow-up of 50 months (range 16–128). The mean elbow flexion of the injured side was 126° (range110–145°) with an average extension loss of 8° (range 0–40°). Pronation was 65° (15–90°) and supination 66° (5–90°). The mean MEPI was 87 points (range 45–100). The mean DASH score was 13 points (range 1–88). According to the Broberg and Morrey functional scoring system, the average score was 92 points (range 88–100). Complete bone union was achieved in 9 cases, partial union in 4 cases and nonunion in one case. There were no signs of avascular necrosis of the radial head. Signs of post-traumatic osteoarthritis were seen in 11 cases. Five patients needed an implant removal due to a radio-ulnar impingement and one patient a revision surgery due to the nonunion and implant breakage.

**Conclusions:**

An on-table (ex situ) reconstruction of the radial head is a reliable option with a good clinical outcome and low complication rate in the surgical treatment of comminuted radial head fractures. It can restore joint alignment and maintain radial length. The risk for avascular necrosis is neglectable, and the bone healing rate is high.

## Background

Fractures to the radial head account for approximately 1.5–4% of all fractures in adults [1]. While undisplaced (Mason type 1) or minimally displaced (by up to 2 mm/Mason type 2) radial head fractures can be treated conservatively, displaced and comminuted radial head fractures (Mason type 3) and elbow-fracture dislocations with simultaneous radial head fractures (Mason type 4) usually need surgical treatment. Depending on the number, size and displacement of all fracture fragments, different surgical treatment options have been described [2–4]. Open reduction and internal fixation (ORIF), arthroplasty or radial head resection are the main surgical options, which lead to different clinical results [3, 5–12].

Formerly, radial head resection was a typical procedure treating unreconstructable radial head fractures [13]. However, it is avoided nowadays, because the radius can migrate proximally and cause distal radio-ulnar joint complaints and reduce elbow joint stability [14]. Arthroplasty can be offered in extremely comminuted cases but is rather seen as a salvage procedure in unreconstructable cases [15]. The first treatment of choice is typically ORIF for Mason-Johnston type 3 fractures. Usually, ORIF is performed in situ to preserve the blood supply to all fracture fragments. However, performing ORIF in situ can be challenging and sometimes not possible due to the comminution and small working space [16, 17].

If the surgeon cannot reconstruct the radial head in situ, he or she has the option to perform an on-table reconstruction of the radial head ex situ [6, 16–18]. There are only small cases series’ [6–9 patients] in the literature reporting about on-table reconstructions of severely comminuted radial head fractures [16–18].

The aim of this study was to enlarge the existing evidence on the outcome of this treatment. We conducted a retrospective study to evaluate the clinical and radiological outcome of patients with an ex situ/on-table reconstructed radial head fracture and compared the results to the existing literature.

## Methods

All patients above the age of 18 who underwent a surgical on-table ORIF treatment for a comminuted and displaced radial head fracture or fracture dislocation in our clinic between 2010 and 2020 were enrolled in this retrospective study. Approval to conduct this follow-up study was obtained from the local ethical review board.

Fourteen consecutive patients (10 males, 4 females, mean age 41.3 years [21–69 years]) were included in this study. Further five patients were either not reachable or not willing to take part in this follow-up study (by phone and mail). Nine included patients sustained a Mason type III and five patients a Mason type IV fracture. An Essex-Lopresti injury was diagnosed in one of the cases. The medial collateral ligament of the elbow was ruptured in seven cases. In 3 cases, the ligament was attached using a transosseous suture technique and in four cases, the ligament was attached using a 2.9 Juggerknot (Zimmer Biomet, Warsaw, Indiana, USA) anchor system. The lateral collateral ligament was ruptured in two cases and reattached using a suture anchor (2.9 Juggerknot Zimmer Biomet, Warsaw, Indiana, USA). The coronoid process was fractured in six cases: five avulsion coronoid fractures (Regan and Morrey type 1) and one Regan and Morrey type 2 fracture which was stabilized using two screws. There was no associated neurovascular injury in any patient. None of the patients had a previous injury to the affected elbow. The indication for an on-table reconstruction was chosen intraoperatively if by all means feasible because of the relatively low patient age and their high claim to elbow.

Primary diagnosis was confirmed by conventional radiographs and CT scans of the elbow.

### Surgical procedure

All patients were treated by two experienced surgeons in our level I trauma center in a supine position under general anesthesia. A lateral Kocher approach was performed with sparing of the collateral ligament if intact. The radial head fracture was assessed intraoperatively, and the decision to perform an on-table reconstruction was based by the intraoperative situation and reconstruction possibility. In all cases, at least 75% of the radial head was completely detached from any periosteal attachments and reconstructed on-table. The radial head fragments were retrieved from the joint and fixed on-table using reduction clamps and 1.0 mm Kirschner wires and/or by interfragmentary screw fixation (2.0 cortical screws [Aptus, Medartis AG, Basel, Switzerland]). To prevent heterotopic ossifications, all fracture fragments were removed from the surgical area including smaller ones, which were attached to the metaphyseal periosteum by a small tissue bridge. After radial head reconstruction, it was reduced and fixed to the radial shaft using temporary placed K-wires and a preshaped radial head plate (Medartis, Basel, Switzerland) was attached in neutral forearm rotation. The plate was placed as laterally as possible to avoid any interference or notching during forearm rotation. However, the plate position was also dictated by the fracture characteristics. First the plate was fixed to the radial head by two angular stable screws, but additional independent lag screws in the head were sometimes necessary depending on the head fragment configuration. Then, the plate was fixed to the shaft by conventional cortex screws. Ruptured ligaments were reattached using an anchor system (Mitek GII anchor system; Johnson and Johnson Co., Switzerland) if needed. The elbow stability and the range of motion (ROM) were checked clinically and fluoroscopically in 0/30/60 degrees of flexion and varus/valgus stress before closure of the surgical approach. In one case, an external fixator was applied promoting for additional stability.

### Postoperative management

The elbow was immobilized in a removable brace for two weeks without motion for wound healing followed by active-assisted exercises in cases of an isolated radial head fracture. In cases of a collateral ligament surgery, a functional brace, with a ROM of 0–40–100°, was applied for 6 weeks to achieve stable ligament healing. Active-assisted exercises (flexion/extension and pronation/supination in the range of 45–0–45°) were started shortly after surgery. Weight bearing was restricted for 6 weeks and full load bearing for 12 weeks after surgery.

### Follow-up examinations

The clinical examinations were performed by a senior physician specialized on injuries of the upper extremity and who was not involved in the previous surgical treatment. The objective clinical measurement parameters were the active range of motion (ROM) of the elbow (extension/flexion and pronation/supination) which was assessed using a standard goniometer, and the grip strength which was assessed using a dynamometer. Grip strength results were compared to the contralateral, uninjured side.

The functional outcome was assessed using the “Disability of the Arm, Shoulder and Hand Score” (DASH), the “Mayo Elbow Performance Index” (MEPI) and the Broberg and Morrey rating system. Pain was measured using the visual analogue score (VAS) while resting and under load with 0 meaning no pain and 10 meaning the most severe pain.

Radiologic follow-up consisted of an anteroposterior and lateral radiograph of the elbow. Radiograph evaluation was conducted by two uninvolved senior physicians. Radiographs were evaluated for nonunions, in-congruencies, loss of reduction, radial head necrosis (dissolving of the radial head), heterotopic ossifications classified according to Hastings and Graham [19], sings of posttraumatic osteoarthritis, implant failure or loosening. Radiographic sings of posttraumatic osteoarthritis were rated according to the Broberg and Morrey classification [20] as grade 0 (normal joint), grade I (minimal joint space narrowing and osteophyte formation), grade II (moderate joint space narrowing and osteophyte formation) and grade III (severe joint space narrowing and osteophyte formation).

Any complications during the healing process (like infections or implant related problems) were recorded from the patient`s records.

The data were de-identified primarily and were recorded and analyzed using Microsoft Excel (Version 2016, Microsoft Corporation, Redmond, Washington, USA). All data were presented using descriptive statistics.

## Results

74% of all treated patients with an on-table reconstruction (14/19) were available for the follow-up examination. The mean follow-up was 50 months (range 16–128; SD 34). The average number of radial head fragments, which were fixed on table, was 4 (range 2–8; SD 2) per patient based on the review of the operation record.

### Clinical outcome

All but three elbows were clinically stable tested in in 0/30/60 degrees of flexion and varus/valgus stress at last follow-up and in anterior–posterior and radiographic findings; however, they did not need any further surgical treatment. The ROM, grip strength, DASH score, MEPI and Broberg and Morrey rating score are shown in Table 1.

**Table 1:** Clinical outcome data (for this case series and existing literature)

	Study design	Number of cases	Number of cases with Mason type III/type IV injuries	Number of cases with collateral ligament injuries	Number of cases with a terrible triad	Number of cases with a Monteggia fracture	Number of cases with fractures of the coronoid process	Mean follow-up in months (range)	Mean age in years (range)	Mean flexion in degrees (range)
Businger et al. [16]	Retrospective case series	6	2/4	3	1	1	2	112 (47–157)	40 (24–48)	141 (136–146)
Kumar et al. [17]	Retrospective case series	6	6/0	3	0	0	0	25 (12–30)	35 (25–46)	135 (125–140)
Everding et al. [18]	Retrospective case series	9	2/7	8	2	4	2	39 (11–64)	47 (22–64)	120 (100–140)
This study	Retrospective case series	14	9/5	7	3	0	6	50 (16–128)	41 (21–69)	126 (110–145)
Cumulative calculations		35	19/16	21	6	5	10	54 (11–157)	41 (21–69	129 (100–146)

	Mean loss of extension in degrees (range)	Mean pronation in degrees (range)	Mean supination in degrees (range)	Grip strength in % of the uninjured wrist (range)	Mayo Elbow Performance Index (range)	Broberg and Morrey Functional Rating Index	Disabilities of Arm, Shoulder and Hand Score	Return to pre-injury occupation/%
Businger et al. [16]	6 (0–15)	79 (68–85)	70 (32–90)	92 (74–104)	99 (95–100)	97 (93–100)	2 (0–6)	4 (+ 1 student)/83%
Kumar et al. [17]	5 (0–10)	70 (65–82)	75 (70–80)	N.s	N.s	90 (75–100)	2 (0–6)	N.s
Everding et al. [18]	20 (0–60)	70 (50–90)	50 (0–90)	N.s	82 (15–100)	N.s	20 (0–85)	9/100%
This study	8 (0–40)	65 (15–90)	66 (5–90)	103 (92—109)	87 (45–100)	92 (88–100)	13 (1–88)	13/93%
Cumulative calculations	10 (0–60)	70 (15–90)	64 (0–90)	99 (74–104)	88 (15–100)	93 (66–100)	11 (0–88)	26 (+ 1 student)/93%

Two patients were pain-free, while 8 patients described mild pain, 3 moderate pain and 1 patient described heavy pain only at the extremes of his active range of motion. All patients but one (who suffered from Parkinson’s disease) returned to their pre-injury occupation.

### Radiologic outcome

There were no signs of avascular necrosis or devitalization of the radial head at radiographical follow-up. Complete fracture union was achieved in 9 cases, partial union in 4 cases and one patient presented an asymptomatic nonunion. Eight patients showed gaps between the fracture fragments of the radial head with a mean gap size of 2.3 mm (range 1–14 mm). Five patients showed fracture steps of the radial head with a mean step size of 0.7 mm (range 1–3 mm). Heterotopic ossifications were found in one patient. Posttraumatic degenerative changes were seen in 11 cases. They were graded as Broberg and Morrey grade 0 in 3 patients, grade I in 6 patients, grade II in 3 patient and grade III in 2 patients. The mean DASH score was 5 for grade 0, 4 for grade 1, 13 for grade 2 and 52 for grade 3.

### Complications

**Fig. 1:**
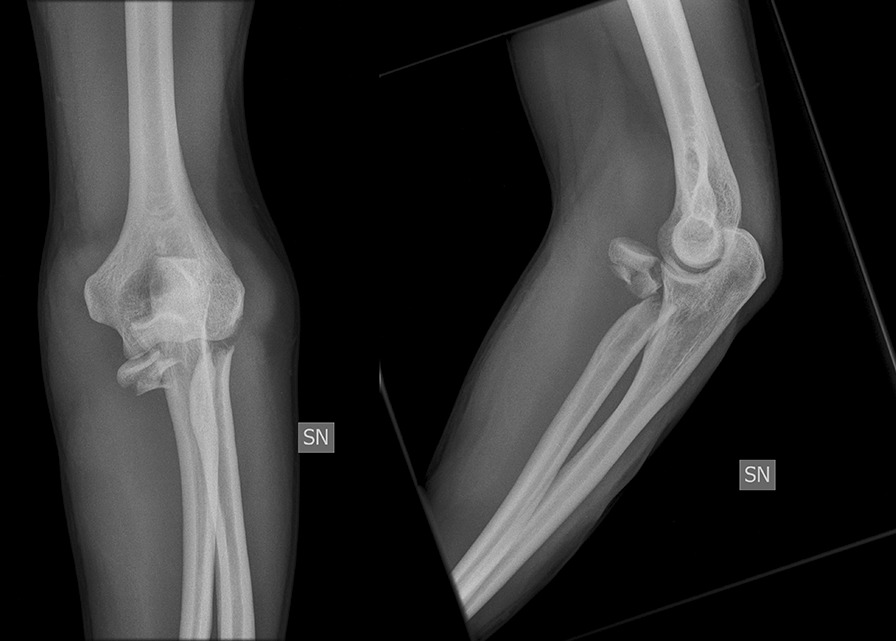
Radiographs of a comminuted radial head fracture

**Fig. 2:**
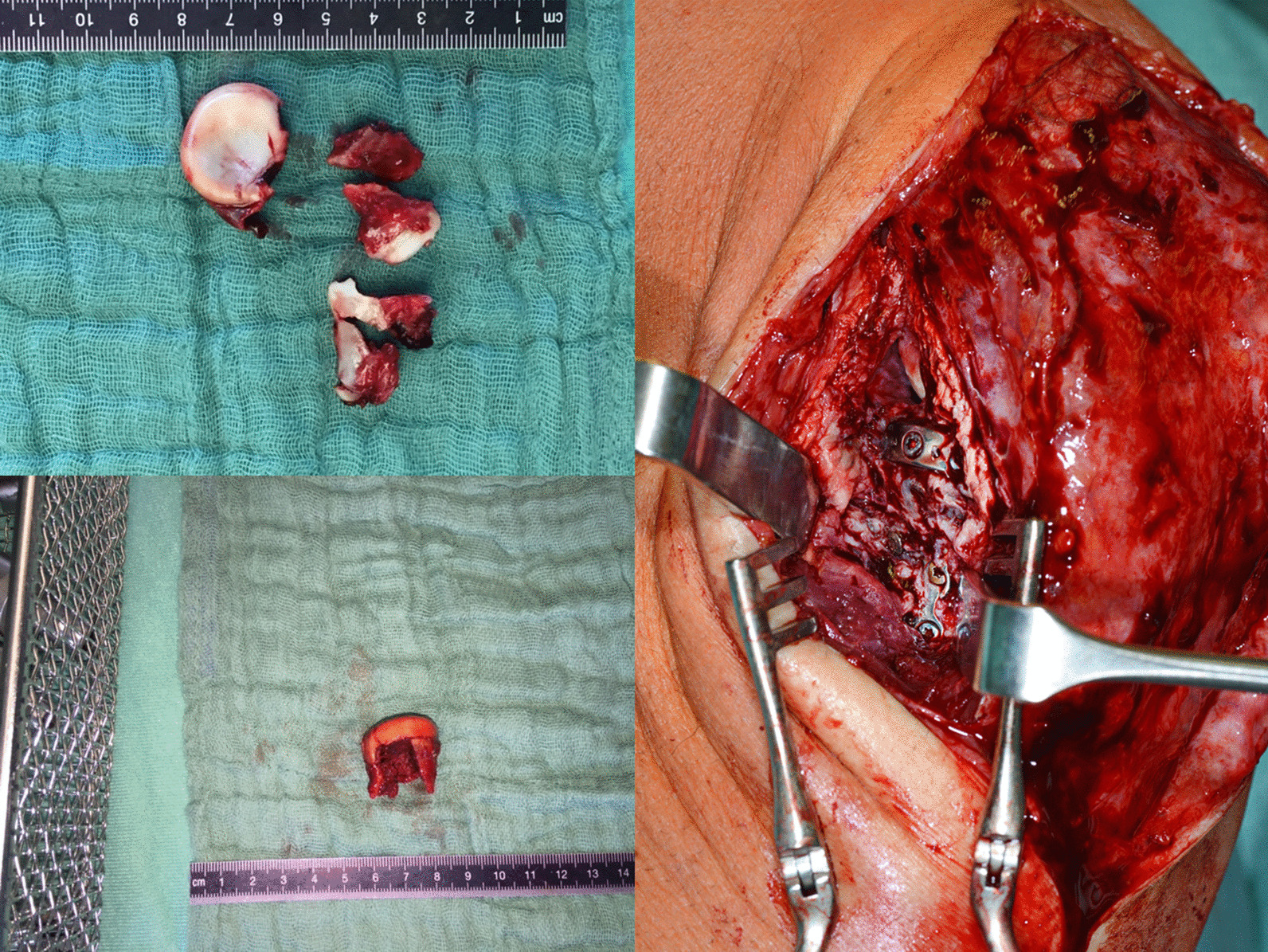
On-table reconstruction of the comminuted radial head

**Fig. 3:**
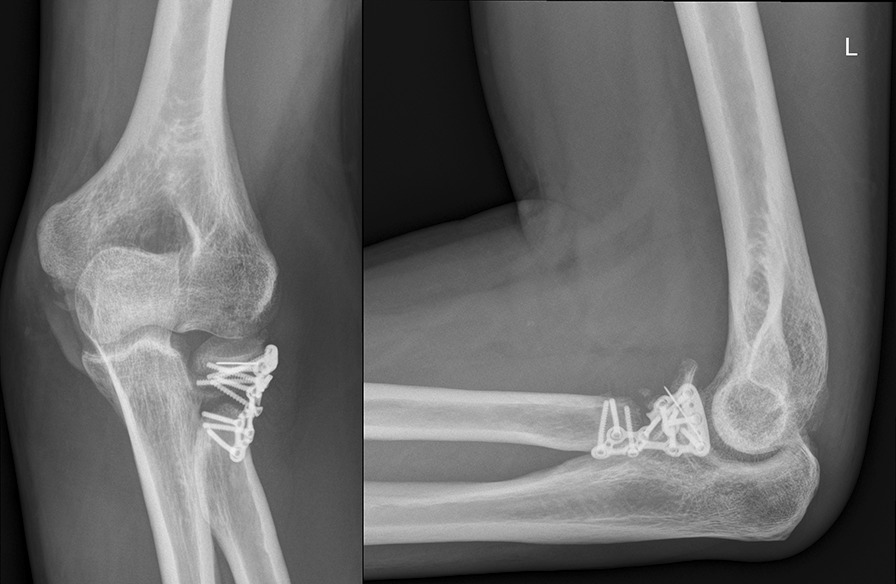
Nonunion of the reconstructed radial head

**Fig. 4:**
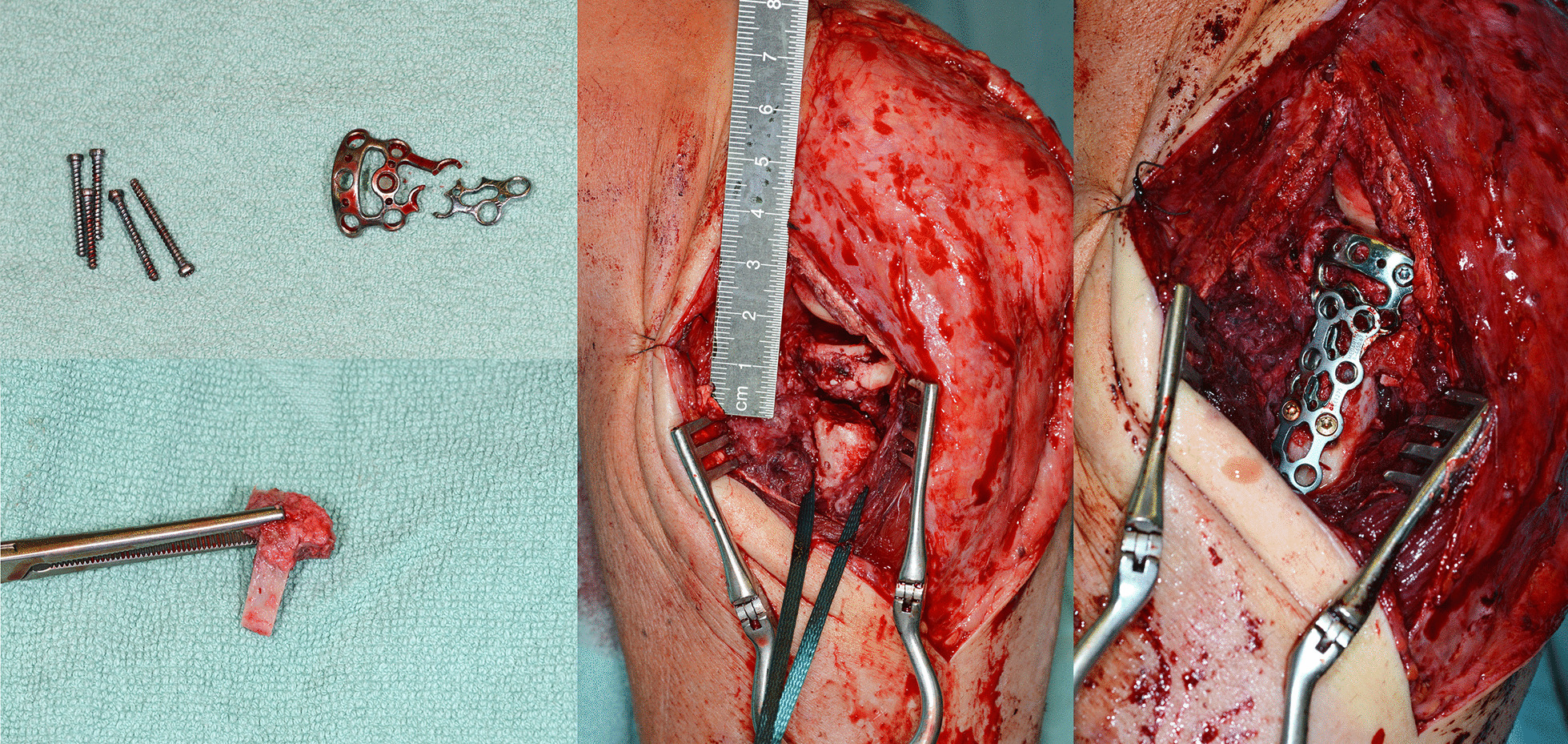
Intraoperative situ showing the implant breakage, additional iliac crest bone graft and re-osteosynthesis with an additional plate

**Fig. 5:**
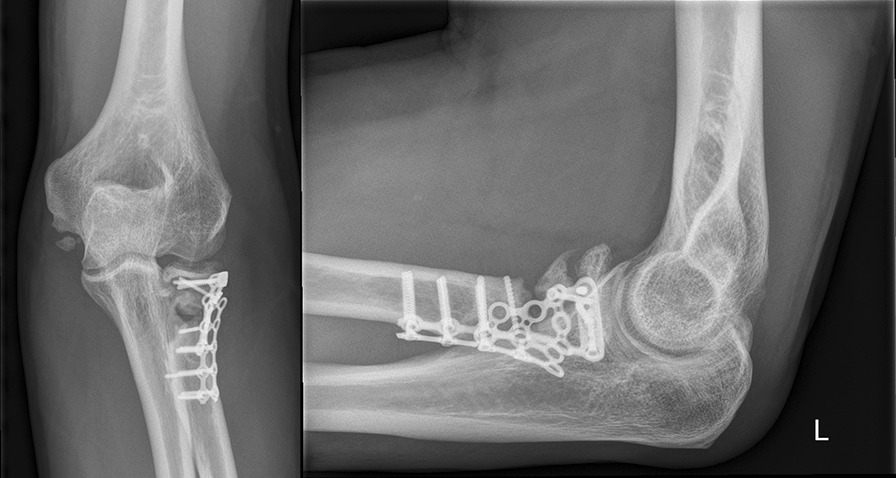
Final radiographs (sagittal range of motion: 0–5–130°; rotational range of motion 65–0–45°; MEPI 85; DASH 16)

The radial head implants had to be removed in 5 cases due to a radio-ulnar impingement with limited forearm rotation and soft tissue irritation after fracture healing. The average time until implant removal was 12.5 months (range 9–14 months). One patient required a revision surgery because of plate breakage 9 months after surgery due to a nonunion without avascular necrosis of the radial head. The revision was performed using another plate to stabilize the fracture that was filled with an additional bone graft harvested from the iliac crest. The final outcome of this patient was rather good with a DASH score of 16.4, a MEPI score of 85 and a range of motion (extension–flexion) of 0–5–130° and a range of motion pro- and supination 65–45° (Figs. 1, 2, 3, 4, 5). A posttraumatic elbow stiffness was treated by open arthrolysis in 4 cases after a mean of 12 months after surgery combined with implant removal. No secondary radial head resection or conversion to arthroplasty had to be performed.

Table 2 shows a summary of postoperative complications.

**Table 2:** Complications outcome data (for this case series and existing literature)

	Number of cases	Non-/Malunions	Avascular necrosis	Elbow instability	Degenerative changes	Hardware breakage	Implant loosening/dislocation	Infections	implant associated soft tissue irritation	Signs of heterotopic ossifications	Neurological deficits	Elbow stiffness	Revision surgery	Pain
Businger et al. [16]	6	0	0	0	1*	N.s	0	0	N.s	N.s	N.s	N.s	N.s	1 (mild)
Kumar et al. [17]	6	3	1**	0	0	0	0	N.s	N.s	0	N.s	N.s	N.s	0
Everding et al. [18]	9	1	0	0	9***	N.s	N.s	N.s	3	9****	0	2	7'	N.s
This study	14	1	0	3	11*****	1	3	0	5	1	0	4	6	2
Cumulative calculations	35	5	1	3	21	1	3	0	8	10	0	6	13	1.7

*According to the Broberg and Morrey Score: 1 × grade I

**Nonunion with avascular necrosis

***According to the Broberg and Morrey Score: 5 × grade I, 4 × grade II

****According to the Hastings and Graham Score: 5 × grade I, 3 × grade IIa, 1 × grade IIb

*****According to the Broberg and Morrey Score: 6 × grade I, 3 × grade II, 2 × grade III

' 2 × implant removal due to ulno-radial impingement; 2 × arthroscopic arthrolysis due to stiffness; 3 × ulnar plate removal due to soft tissue irritation

## Discussion

Displaced and comminuted radial head fractures are complicated injuries and difficult to treat. This case series showed that a radial head reconstruction ex situ is a viable and good option as it leads to a good clinical outcome. Cumulative calculations showed that 93% of patients can expect to return to their pre-injury occupation. Although some patients will suffer a minor extension loss (mean 10°), this does not affect their functional outcome significantly, as the mean MEPI was 89, the mean Broberg and Morrey rating index 90 and the mean DASH score 11. This case series showed similar clinical and functional outcome results to previous reports on on-table reconstructions (Table 1) [16–18], as well as on Mason type 3 and 4 ORIF procedures [3, 10, 12, 21].

Another major finding of these studies and ours is that although the radial head is reconstructed ex situ without any blood supply, the rate of avascular necrosis is low. Most patients showed a healed radial head fracture. Only one patient showed an avascular necrosis. However, the patient did not develop pain, crepitus or implant breakage and no additional surgical intervention had to be performed [17]. The nonunion rate seems to be high [5/35 patients (Table 2)]; however, the need for subsequent surgical procedures was low because most patients were asymptomatic (3 patients). This leads to the assumption that these cases are more or less stable fibrous nonunions. In our series, only one patient had to be revised because of an implant breakage. In general, it seems that a radiologic nonunion of the radial head does not need a revision surgery possibly due to a fibrous nonunion and low force transfer over the radial column to the capitulum [22].

One important downside is that 4 out of 12 patients needed an implant removal because of implant soft tissue irritation or radio-ulnar impingement with a loss of forearm rotation (Table 2). To avoid rotational limitations and a radio-ulnar impingement, the plate should be positioned in neutral position of the forearm on the lateral side of the proximal radius in the so-called safe zone [23]. However, perfect plate placement cannot always be achieved because of the fracture pattern and/or screws, which were used for on-table head fixation. Additionally, limited anatomical space makes the ORIF procedure difficult especially by increasing numbers of fracture fragments and the loss of periosteal attachment [17]. The use of anatomical preshaped implants is advantageous because there are more options for the use of angular stable screws.

Despite the good clinical outcome, eleven patients showed some degenerative changes; however, most of them were minor grade 1 changes (Table 2). Regarding the low pain score, most of these changes were mainly asymptomatic. Three patients with a Broberg and Morrey grades 0 and 6 patients grade 1 had a mean pain score of 1.8.

Two other treatment options for comminuted, unreconstructable types of fractures include radial head excision and primary arthroplasty. The role of the radial head as a secondary stabilizer of the forearm and elbow was described by Ring et al. [24]. The radial head is an important stabilizer of the elbow joint. Combined with the medial collateral ligament, it stabilizes the elbow joint against valgus stress. The radial head is also involved in longitudinal stability of the elbow and the forearm and plays an important role for posterolateral rotatory stability together with the coronoid process. Problems of radial head excision include weakness, pain, instability, decreased strength, osteoarthritis, a subsequent cubitus valgus deformity and proximal radial migration/translation [5, 18, 25–28]. Despite these problems, Antuna et al. reported a mean MEPS of 95 points with a mean ROM (extension–flexion) of mean 9°–139° and a mean pronation of 84° and a supination of 85° in a study of 26 patients who were retrospectively reviewed after a minimum of 15 years (mean 25 years) after a primary radial head resection [29]. The mean grip strength was 39 kg compared to 45 kg of the non-injured side (87%) [29]. However, radiocapitellar contact seems to play a major role in the prevention of the development of ulnohumeral osteoarthritis, weakness and elbow instability [21, 26, 30, 31]. Therefore, radial head excision is not recommended nowadays as a primary solution and should not be performed [17, 18].

Taking into account the radial head as a secondary stabilizer, the aim of a radial head prosthesis is the reconstruction of the radial column to assure physiological force transmission from the forearm to the humerus. Ring et al. [24] recommended that radial head fractures of more than 3 parts should be treated using radial head prosthesis or radial head resection because the results of reconstructed radial heads lead in 54% of the involved patients to an unsatisfying results (3× failure of fixation which lead to radial head excision, 6× painful nonunion which lead to radial head excision, 4× limitation of forearm rotation lesser than 100°).

Heijink et al. [32] report in a systematic review on radial head arthroplasty. The range of motion after radial head arthroplasty was 0–15–130° with a postoperative flexion–extension arc of a mean 115°, and the mean pronation–supination was 70–0–72° with a pronation–supination arc of a mean 140°. These results for the ROM seem similar to the results of an ex situ ORIF of the radial head.

However, a retrospective comparison between arthroplasty and ORIF showed a higher stiffness in the ORIF group with a sagittal range of motion of 0–30–110° for ORIF and 0–21–124° for arthroplasty, and with a rotational range of motion of 51–0–61° for ORIF and 68–0–77° for arthroplasty. Yet, functional scores did not show any difference between both groups (DASH 29 vs. 29; Broberg–Morrey score 75 vs. 77 and MEPI 84 vs. 86) [33]. A better forearm rotation was also seen by other authors compared to our results and by other authors for radial head arthroplasty. In that study, sagittal and rotational range of motion was reduced by about 10° compared to the contralateral side after arthroplasty. Although the median range of motion was 0–0–138°, 8% of patients showed an extension lag of over 30°. Additionally, 55% of patients reported on a subjective loss of strength with a grip strength of 89% compared to the contralateral side [34].

Regarding the complication rate, ORIF showed a higher complication rate in a direct retrospective analysis compared to arthroplasty (50% vs. 20%). The main reasons for revision surgery after ORIF were secondary displacement, nonunion and hardware breakage [33]. On the other hand, other authors found no difference in the short-term (30-day) perioperative surgical and medical complication rate between arthroplasty and ORIF evaluating 435 patients [35]. Revisions and reoperations after radial head arthroplasty occur mainly within the first three years with a cumulative probability at years 3 years following arthroplasty of 6.5% for revision and 8.2% for reoperation [36]. The five-year implant survival for arthroplasty ranges between 71 and 100% with a similar long-term survival rate [37–41].

In our opinion, radial head arthroplasty is a reliable option if stable fixation using ORIF cannot be achieved intraoperatively. The on-table procedure should be performed whenever possible, if anatomical reconstruction of the head and stable fixation to the neck can be achieved in younger patients with a good bone stock. A radial head arthroplasty can still be conducted as a salvage procedure in failed ORIF cases [6]. Current literature shows a lack of randomized, controlled trials for comparing radial head on-table outcomes with other procedures.

Although this study presents the highest patient count that was treated with an on-table radial head reconstruction, the main limitation of this and previous studies is still the low number of patients and the loss to follow-up of 26%. However, this injury is quite rare and a significantly higher count can only be achieved in future work with multicenter studies. Additionally, comparisons between arthroplasty and ORIF can only be conducted in a larger multicenter study. The retrospective character is a limitation by its nature. Potential outcome parameters like forearm rotation strength or elbow flexion and extension strength were not assessed in this study or previous studies, which may be worth investigating at last; degenerative changes may increase in time and become symptomatic, which could not be assessed with a reasonable mid-term follow-up of 50 months.

## Conclusions

An on-table reconstruction and fixation of comminuted radial head fractures using a low profile plate is a reasonable and safe option and leads to a good clinical outcome with a good joint mobility and with a low complication rate. The reconstructed radial head acts as spacer and adds stability even if an asymptomatic nonunion is seen radiologically. In comparison with existing literature, we conclude that the risk of avascular necrosis after complex, comminuted radial head fractures and an ex situ/on-table reconstruction is very low.

## Data Availability

The data can be obtained from Dr. Tobias Kastenberger.
